# Myc inhibition impairs autophagosome formation

**DOI:** 10.1093/hmg/ddt381

**Published:** 2013-08-09

**Authors:** Pearl P. C. Toh, Shouqing Luo, Fiona M. Menzies, Tamás Raskó, Erich E. Wanker, David C. Rubinsztein

**Affiliations:** 1 Department of Medical Genetics, University of Cambridge, Cambridge Institute for Medical Research, Addenbrooke's Hospital, Hills Road, Cambridge CB2 0XY, UK; 2 Max Delbrueck Center for Molecular Medicine, Robert-Roessle-Str. 10, Berlin Buch D-13125, Germany

## Abstract

Autophagy, a major clearance route for many long-lived proteins and organelles, has long been implicated in cancer development. Myc is a proto-oncogene often found to be deregulated in many cancers, and thus is an attractive target for design of cancer therapy. Therefore, understanding the relationship between anti-Myc strategies and autophagy will be important for development of effective therapy. Here, we show that Myc depletion inhibits autophagosome formation and impairs clearance of autophagy substrates. Myc suppression has an inhibitory effect on autophagy via reduction of c-Jun N-terminal kinase 1 (JNK1) and B-cell lymphoma 2 (Bcl2) phosphorylation. Additionally, the decrease in JNK1 phosphorylation observed with Myc knockdown is associated with a reduction in ROS production. Our data suggest that targeting Myc in cancer therapy might have the additional benefit of inhibiting autophagy in the case of therapy resistance associated with chemotherapy-induced autophagy.

## INTRODUCTION

Macroautophagy (hereafter called autophagy) is an evolutionarily conserved process for bulk degradation of cytoplasmic contents. It involves the sequestration of portions of cytoplasm through the formation of double-membraned autophagosomes, which are subsequently targeted for degradation after fusion with lysosomes. Under normal conditions, autophagy is maintained at a basal level (1), where it is essential for quality control of the cytoplasmic environment through the maintenance of energy balance and by eliminating dysfunctional organelles and aggregate-prone proteins. Under stress conditions, such as starvation and hypoxia, autophagy is induced as an adaptive response, and drives the catabolism of proteins, lipids and carbohydrates for *de novo* biosynthesis of biomacromolecules, which enables cells to meet their metabolic and energy demands (2). Therefore, autophagy is crucial for conferring cytoprotective effects and promoting cell survival under various stress conditions.

Autophagy can be regulated via diverse pathways. For instance, amino acid depletion can induce autophagy by inhibiting the mammalian target of rapamycin (mTOR) (3). There are also a number of mTOR-independent autophagy pathways (4,5). In one of these, nutrient depletion leads to increased JNK1 activity, which phosphorylates B-cell lymphoma 2 (Bcl2) (6). This phosphorylation decreases the inhibitory interaction of Bcl2 with the core autophagy protein, Beclin 1, thereby increasing Beclin 1 activity. This, in turn, activates autophagy, since Beclin 1 activates the lipid kinase Vps34, vacuolar protein sorting 34, which generates phosphatidylinositol 3-phosphate to enhance autophagosome formation.

Dysregulation of autophagy can lead to malfunction of cellular processes and thus, contribute to pathological conditions, including neurodegenerative disorders, metabolic diseases and cancer. In fact, cancer was the first human pathology associated with autophagy, as revealed by the discovery that expression of Beclin 1 was down regulated in 40–75% of human breast and ovarian cancers due to monoallelic deletion of the gene (7). In addition, deletion of various regulators of autophagy, including Bax interacting factor-1 (*Bif-1*), ultraviolet radiation resistance-associated gene (*UVRAG*) and *Atg4*, has been shown to promote the development of spontaneous tumours in mice (8,9). These studies indicate that autophagy may protect against cancer development.

On the other hand, by virtue of its pro-survival function, autophagy has also been suggested to contribute to cancer progression, especially in the late stages of disease. In particular, tumour cells growing in poorly vascularized regions are deprived of nutrients and oxygen for extended periods of time. Activation of autophagy enables recycling of macromolecules and organelles, thereby accommodating the acute energy needs of cancer cells (10,11). Furthermore, autophagy has been proposed as a common mechanism mediating therapeutic resistance to various cancer therapies (12,13). Therefore, the role of autophagy in tumourigenesis appears to be context-dependent and dynamic with the evolution of tumour cells during the course of cancer progression.

Cancer encompasses a group of diseases resulting from genetic changes that lead to unregulated cell proliferation and eventually invasive transformation. Deregulated expression of c-Myc (hereafter referred to as Myc) is one of the most common lesions occurring in human cancers of diverse origin (14,15), as high levels of Myc can lead to uncontrolled cell expansion and malignant transformation (16). There is also emerging evidence that established tumour cells may acquire a dependency on Myc activity (17), and thus, inhibition of Myc could represent a novel therapeutic strategy for Myc-induced cancers. Indeed, Myc depletion by using small-interfering RNA (siRNA), antisense oligonucleotides or phosphorodiamidate morpholino oligomers (PMOs) has been shown to trigger cell growth arrest and apoptosis in transformed cells as well as reduced growth of tumour xenografts (18,19). Similar observations were made in *in vivo* studies, in which inhibition of Myc in a preclinical murine model of lung adenocarcinoma induced apoptosis, breakdown of the tumour microenvironment, followed by rapid tumour regression (20). These data have led to the development of promising strategies to allow down regulation of Myc for cancer therapy, including the development of transcriptional repressors (21).

In situations where Myc levels were elevated, autophagy appeared to be decreased (22,23). This suggests that inactivation of Myc will lead to an increase in autophagy, which may be less than optimal for treating existing cancers. Therefore, understanding the effects of Myc depletion on autophagy is relevant for the design of anti-neoplastic therapy, as autophagy modulators and inhibitors are currently being investigated as chemotherapy strategies. Accordingly, we sought to understand the effects of Myc suppression on autophagy. Contrary to our expectations, we showed that Myc knockdown or inhibition had a very clear inhibitory effect on autophagy. We further elucidated that Myc regulates autophagy through changes in JNK1 activity and phosphorylation of its downstream target, Bcl2. As conventional cancer treatment often leads to development of resistance towards therapy, it is intriguing to envisage Myc as a target in which the issue of therapy efficacy and resistance can be simultaneously addressed.

## RESULTS

### Myc inhibition impairs autophagosome formation

To investigate whether Myc plays a role in autophagy, we first performed siRNA knockdown of the gene and examined its effects on microtubule associated protein 1 light chain 3 (LC3-II) protein levels. LC3-II is the only known protein to specifically associate with autophagosomes, and thus, it is widely accepted as a marker for monitoring autophagy (24). LC3 is first synthesized as a precursor in the form of pro-LC3. It is then proteolytically processed into LC3-I, which remains as a cytoplasmic protein and does not associate with autophagosomes. LC3-I is subsequently conjugated with phosphatidylethanolamine on autophagosomes to form LC3-II. LC3-II levels robustly correlate with autophagosome numbers (24). The most common method to assay for autophagy is by immunoblotting for LC3-II levels, normalized to actin or tubulin as the loading control (25).

**Figure 1.**
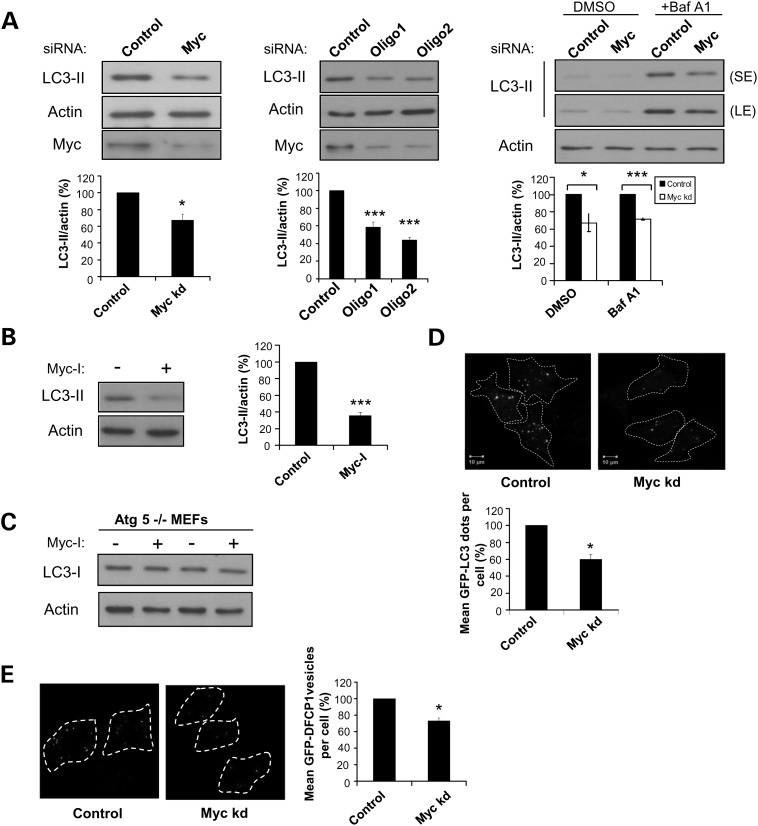
MYC RNAi knockdown decreases LC3-II levels. (**A**) (Left panel) HeLa cells subjected to control or Myc siRNA knockdown for 72 h were lysed and analysed by western blotting. Western blot shows the knockdown efficiency of Myc protein. Graph shows quantification of the LC3-II/actin ratio. Endogenous LC3-II was quantified by densitometry analysis and normalized to actin. Relative protein levels were expressed as a percentage relative to the control cells, which were set to 100. (Middle panel) HeLa cells subjected to control or deconvoluted Myc oligo (oligos 1 and 2) siRNA knockdown for 72 h were lysed and analysed by western blotting. Western blot shows the knockdown efficiency of Myc protein. Graph shows quantification of the LC3-II/actin ratio. (Right panel) HeLa cells subjected to control or Myc siRNA knockdown for 72 h were treated with 400 nm bafilomycin A1 (Baf A1) or DMSO during the last 4 h and quantified as above. Graph shows quantification of the LC3-II/actin ratio. SE, short exposure; LE, long exposure. (**B**) HeLa cells treated with 60 µm Myc inhibitor (Myc-I) for 16 h were assessed for LC3-II levels by western blotting. Graph shows quantification of the LC3-II/actin ratio. (**A** and **B**) The values represent the mean ± standard error of the mean (SEM) of the percentage of LC3-II/actin from three independent experiments. Data for comparison between the two groups were analysed by using a two-tailed *t*-test as described in Methods section, *P < 0.05; ***P < 0.0005. Statistical significance for comparison between more than two groups was analysed by one-way ANOVA, with Dunnett post hoc test. (**C**) Atg5−/− MEFs treated with 60 µm Myc inhibitor (Myc-I) for 16 h were assessed for LC3-I levels by western blotting. (**D**) HeLa cells with stable expression of GFP-LC3 were subjected to control or Myc siRNA knockdown for 72 h and fixed for quantification of LC3 dots. The number of LC3 dots was quantified by automated counting (see the Methods section). Representative images of the GFP-LC3 cells are shown. Graph shows quantification of the percentage of mean GFP-LC3 dots per cell relative to the control cells, which were set to 100. At least 500 cells were counted per sample and the values represent the mean ± standard error of the mean (SEM) of the percentage of mean GFP-LC3 dots per cell from three independent experiments. *P < 0.05. Scale bar, 10 µm. (**E**) Hek293 cells with stable expression of GFP-DFCP1 were transfected with control or Myc siRNA for 72 h and fixed for counting of GFP-DFCP1 vesicles under a confocal microscope. Representative images of the GFP-DFCP1 cells are shown. Graph shows quantification of the percentage of mean GFP-DFCP1 vesicles per cell relative to the control cells, which were set to 100. At least 100 cells were counted per sample and the values represent the mean ± SEM of the percentage of mean GFP-DFCP1 vesicles per cell from three independent experiments. *P < 0.05.

Knockdown of Myc in HeLa cells decreased LC3-II levels, and this correlated with a reduction in endogenous Myc expression (Fig. 1A). To verify that this was not due to an off-target effect of the smartpool siRNAs, we confirmed that LC3-II levels were also decreased with two different oligos targeted against Myc (oligos 1 and 2) (Fig. 1B). Since changes in LC3-II levels may reflect alterations in either its rate of synthesis or degradation, LC3-II levels were assessed in the presence of saturating concentrations of Bafilomycin A1 (Baf A1). Baf A1 is a lysosomal inhibitor that blocks the fusion of autophagosomes to lysosomes, thereby preventing LC3-II degradation (26). This allows for specific evaluation of changes in LC3-II formation, without confounding changes in its degradation (25). Myc knockdown decreased LC3-II levels even in the presence of Baf A1, confirming that Myc affects autophagosome formation rather than its degradation (Fig. 1C). Similar observations were obtained in HEK293 cells (Supplementary Material, Fig. S1A) and Myc knockout (−/−) rat fibroblasts (Supplementary Material, Fig. S1B), in which Myc depletion decreased LC3-II levels in both the absence and the presence of Baf A1.

We went on to test a small molecule inhibitor of Myc, 10058-F4 (referred to as Myc-I, Myc inhibitor (10058-F4), hereafter). This compound appears to inhibit Myc both by disrupting its dimerization with its obligate partner, Max, which is required for transactivation of target genes and by reducing Myc mRNA expression in different cell lines (27). Consistent with the genetic data, chemical inhibition of Myc resulted in decreased LC3-II levels in both HeLa (Fig. 1D) and HEK293 cells (Supplementary Material, Fig. S1C).

To exclude the possibility that the decrease in LC3-II levels observed is due to suppression in the transcriptional activity of Myc on LC3, Atg5 knockout (−/−) murine embryonic fibroblast (MEFs) were used. Atg5 −/− MEFs lack the Atg5 protein that is required for the conversion of LC3-I to LC3-II, and thus, only LC3-I can be detected in Atg5−/− MEFs. If there was less transcription of LC3 itself, then lower LC3-I levels would be detected in Atg5−/− MEFs upon Myc inhibition. We observed no change in LC3-I levels in Atg5−/− MEFs treated with an Myc inhibitor, suggesting that Myc inhibition does not result in reduced transcription of LC3 (Fig. 1E).

In addition to immunoblotting, an alternative approach to assess autophagy is by immunofluorescence. For this, we used HeLa cells stably expressing GFP-LC3, in which GFP-LC3 associated with autophagosomes appears as green puncta. We observed that Myc knockdown decreased the number of GFP-LC3 puncta (Fig. 1F).

We also assessed the numbers of double FYVE (zinc finger domain named after four cysteine-rich proteins: **F**ab 1 (yeast orthologue of PIKfyve), **Y**OTB, **V**ac 1 (vesicle transport protein), and **E**EA1) domain-containing protein (DFCP1) vesicles. DFCP1 labels phosphatidylinositol-3-phosphate (PI3P) on autophagosome precursors, called omegasomes (28). Consistent with an inhibition of autophagosome formation, Myc knockdown decreased the mean number of GFP-DFCP1 vesicles per cell in HEK293 (Fig. 1E).

### Myc inhibition causes autophagy substrate accumulation

**Figure 2.**
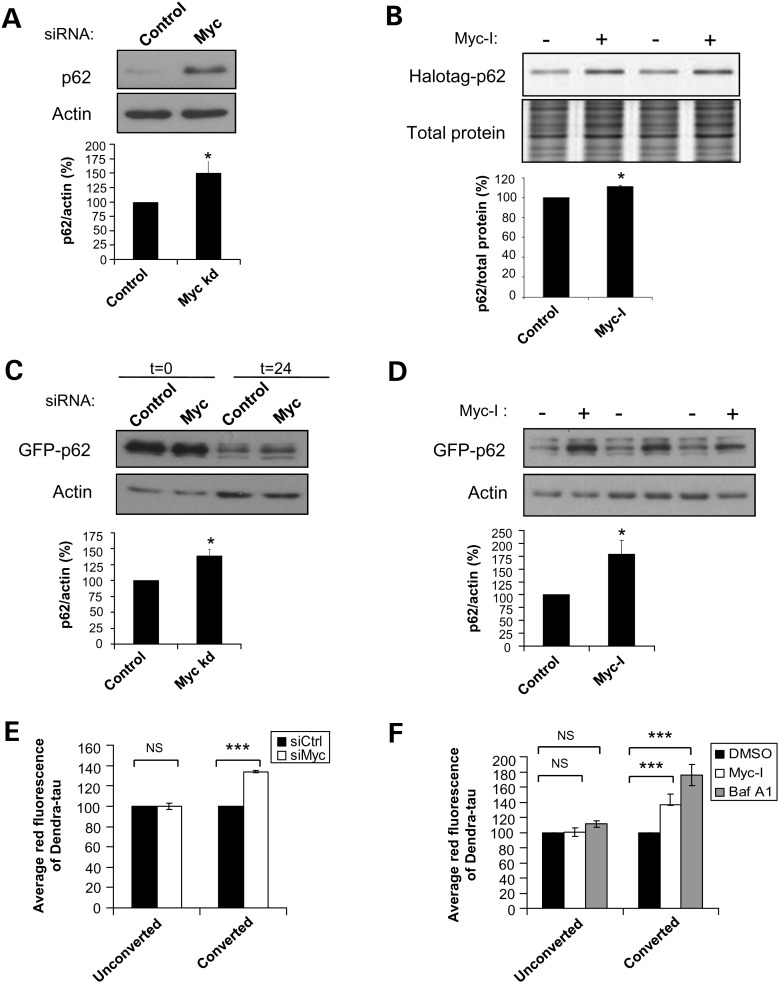
Suppression of MYC blocks clearance of autophagy substrates. (**A**) HeLa cells subjected to control or Myc siRNA knockdown for 72 h were assessed for p62 levels by western blotting. Graph shows quantification of p62/actin ratio. The values represent the mean ± SEM. *P < 0.05. (**B**) U2OS cells with stable expression of Halotag-p62 were labelled with ligand then treated with 60 µm Myc-I or DMSO for 16 h. Cells were lysed and assessed for Halotag-p62 levels by SDS-PAGE relative to the expression of total protein. Graph shows quantification of labelled p62/total protein ratio. The values represent the mean ± SEM of the percentage of labelled p62/total protein from a triplicate experiment representative of three independent studies. **P < 0.005. (**C**) Hek293 cells with tetracycline-inducible expression of GFP-p62 were subjected to control or Myc siRNA knockdown for 72 h. Expression of GFP-p62 was induced with tetracycline 48 h after transfection and ‘turned off’ after 24 h. Cells were collected at the time of ‘turn-off’ (*t* = 0) and 24 h after ‘turn-off’ (*t* = 24) to assess for clearance of GFP-p62 by western blotting. *P < 0.05. (**D**) Hek293 cells with tetracycline-inducible expression of GFP-p62 was induced with tetracyclin and ‘turned off’ after 24 h, followed by treatment with 60 µm Myc-I or DMSO for 16 h. Cells were assessed for clearance of GFP-p62 by western blotting. *P < 0.05. (**E** and **F**) HeLa cells were transiently transfected with tau recombinant constructs containing the Dendra tag. After 24 h, Dendra-tau expressed in the cells were either converted by exposure to 405 nm light source for 2 min or left unconverted. (**E**) Both converted and unconverted cells were subjected to control or Myc siRNA knockdown for 48 h. Cells were fixed and assessed for clearance of Dendra-tau by FACS analysis. Graph shows the average of the mean red fluorescence of three independent studies. (**F**) Both converted and unconverted cells were treated with 60 µm Myc-I, 200 nm Baf A1 or DMSO for 16 h. Cells were fixed and assessed for clearance of Dendra-tau by FACS analysis. Graph shows the average of the mean red fluorescence of three independent studies. *P < 0.05; ***P < 0.0005.

Having established that Myc inhibition impairs autophagosome formation, we next assessed the functional effects of this on autophagy substrate accumulation. p62 (SQSTM1/sequestome1) is a widely characterized endogenous autophagy substrate (29,30). Consistent with a decrease in LC3-II levels, Myc knockdown in HeLa cells increased p62 levels (Fig. 2A). p62 levels were also elevated in Myc −/− rat fibroblasts, compared with wild-type (WT) controls (Supplementary Material, Fig. S2). To validate that this was not predominantly a consequence of Myc on p62 transcription, U2OS cells stably expressing Halotag-p62 were used. The cells were labelled with a Halotag fluorescent ligand before they were treated with Myc-I. The Halotag ligand forms a covalent bond and binds irreversibly to the Halotag protein. This enables a fixed pool of Halotag-p62 protein to be labelled and its levels can be monitored over time after labelling in a chase period which is independent of any changes in protein synthesis (31). Inhibition of Myc during the chase period resulted in more labelled Halotag-p62 levels remaining in comparison to controls, consistent with decreased clearance and autophagy impairment (Fig. 2B). To further confirm if the accumulation of p62 was due to attenuation in its clearance rate, HEK293 cells with inducible expression of GFP-p62 were subjected to knockdown experiments with Myc or control siRNAs. After siRNA transfection, the expression of GFP-p62 was induced for 24 h before being turned off and we assessed the amount of GFP-p62 that was cleared over time, at the time its expression was turned off (*t* = 0), and 24 h after switching-off (*t* = 24). Myc knockdown caused a reduction in the degradation rate of GFP-p62 over time, as evident by the ratio of GFP-p62 remaining at *t* = 24 relative to *t* = 0 (Fig. 2C). Similarly, impaired GFP-p62 clearance was also observed with Myc-I (Fig. 2D).

As p62 expression might be affected by various factors that are unrelated to autophagy (32), we also assessed other proteins that are known autophagy substrates such as tau (33). For this purpose, complementary DNA (cDNA) constructs encoding tau fused with a photo-switchable fluorescent protein, Dendra, were transiently transfected into HeLa cells. Dendra tags can be irreversibly photo-converted from green to red fluorescence by exposure to 405 nm light source. Converted forms of Dendra-tau serve as a fixed pool of substrate proteins that can be monitored over time after photo-switching. Myc knockdown slowed the degradation of converted Dendra-tau, further confirming the data obtained with p62 degradation (Fig. 2E). Likewise, treatment with Myc-I delayed clearance of converted Dendra-tau relative to controls. A similar effect was observed with Baf A1-treated cells (positive control), which blocks degradation of autophagosomes (Fig. 2F). Taken together, these findings demonstrate that Myc inhibition resulted in decreased clearance of different types of autophagy substrates, consistent with impairment in autophagy.

### Myc inhibition affects autophagy via the JNK1-Bcl2 pathway

**Figure 3.**
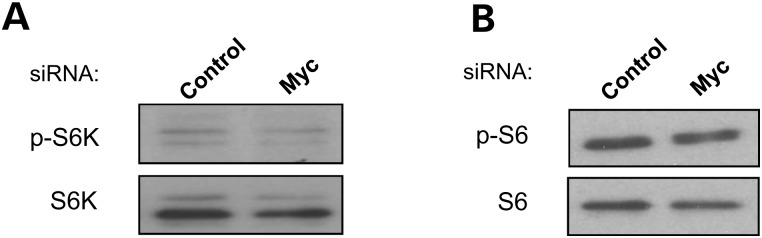
MYC effects on autophagy are independent of mTOR. HeLa cells subjected to control or Myc siRNA knockdown for 72 h were assessed for (**A**) phospho- and total S6K; (**B**) phospho- and total S6 levels by western blotting.

To investigate the molecular mechanism by which Myc modulates autophagy, we first examined the effect of Myc knockdown on mTOR activity, which negatively regulates autophagy. The activity of mTOR can be assayed by measuring the phosphorylation its downstream targets, such as p70S6 kinase or S6 ribosomal protein (1). Myc knockdown did not significantly change the phosphorylation of p70S6 kinase (Fig. 3A) and S6 ribosomal protein (Fig. 3B), indicating that there are other pathways independent of mTOR mediating the effects of Myc on autophagy.

**Figure 4.**
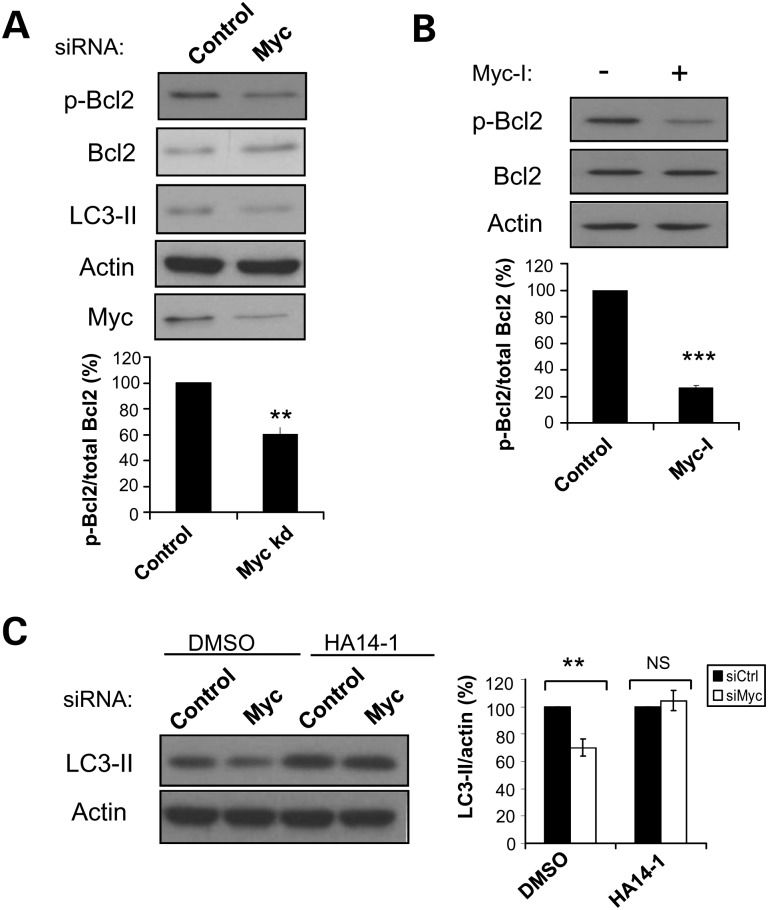
MYC knockdown decreases LC3-II levels via a reduction of Bcl2 phosphorylation. (**A**) HeLa cells subjected to control or Myc siRNA knockdown for 72 h were assessed for phospho-Bcl2 levels by western blotting. (**B**) HeLa cells treated with 60 µm Myc-I for 8 h were assessed for phospho-Bcl2 levels by western blotting. (**A** and **B**) Graphs show quantification of the phospho-Bcl2/total Bcl2 ratio. The values represent the mean ± SEM of the percentage of phospho-Bcl2/total Bcl2 from three independent experiments. **P < 0.005; ***P < 0.0005. (**C**) HeLa cells subjected to control or Myc siRNA knockdown for 72 h were treated with 20 µm HA14–1, a Bcl2 inhibitor or DMSO for the last 16 h. Graph shows quantification of the LC3-II/actin ratio. The values represent the mean ± SEM of the percentage of LC3-II/actin from three independent experiments. **P < 0.005; NS, not significant.

To understand the link between Myc-modulated autophagy and cancer, we tested whether the compromise of autophagy resulting from Myc inhibition was mediated through changes in Bcl2, since the unphosphorylated form of this protein inhibits Beclin 1 to decrease the production of PI3P, which would manifest in decreased DFCP1 vesicles that was observed earlier (Fig. 1F). Myc knockdown and Myc-I decreased phospho-Bcl2, consistent with an impairment in autophagy (Fig. 4A and B). To verify that the effect of Myc-I (10058-F4) on Bcl2 phosphorylation was not due to an unspecific effect of the drug that is unrelated to autophagy, another Myc inhibitor (10074-G5) was used. 10074-G5 targets a region of Myc that is different from Myc-I (10058-F4). We confirmed that 10074-G5 also decreased Bcl2 phosphorylation, in parallel with decreased LC3-II levels (Supplementary Material, Fig. S3).

The effect of Myc knockdown on LC3-II was abrogated when Bcl2 was inhibited with HA14-1, a BH3 mimetic that interferes with the inhibitory Bcl2–Beclin 1 interaction, and thus, would be expected to mimic the functional effects of Bcl2 phosphorylation in this context (34). This rescued the decrease in autophagy that would otherwise be expected with Myc knockdown, suggesting that Bcl2 is downstream of Myc (Fig. 4C). In conclusion, these data suggest that the effects of Myc depletion on autophagy were mediated through changes in Bcl2 phosphorylation.

**Figure 5.**
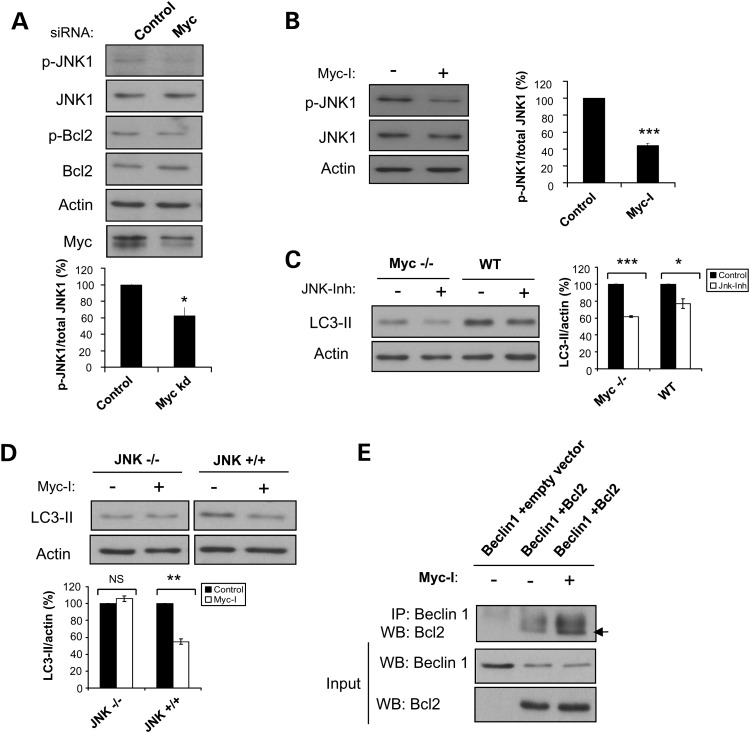
MYC knockdown decreases Bcl2 activity via a decrease in JNK activity. (**A**) HeLa cells subjected to control or Myc siRNA knockdown for 72 h were probed with the indicated antibodies by western blotting. (**B**) HeLa cells treated with 60 µm Myc-I for 8 h were assessed for phospho-JNK levels by western blotting. (**A** and **B**) Graphs show quantification of the phospho-JNK/total JNK ratio. The values represent mean ± SEM of the percentage of phospho-JNK/total JNK from three independent experiments. *P < 0.05; ***P < 0.0005. (**C**) Myc+/+ (WT) or Myc−/−rat fibroblasts treated with 20 µm JNK inhibitor VIII (JNK-Inh) for 3 h were assessed for LC3-II levels by western blotting. (**D**) JNK+/+ or JNK−/− MEFs treated with 60 µm Myc-I for 16 h were assessed for LC3-II levels by western blotting. (**C** and **D**) Graphs show quantification of the LC3-II/actin ratio. The values represent mean ± SEM of the percentage of the LC3-II/actin ratio from three independent experiments. *P < 0.05; **P < 0.005; ***P < 0.0005; NS, not significant. (**E**) HeLa cells co-transfected with Beclin 1 and Bcl2 or empty vector expression constructs for 24 h were treated with DMSO (control) or 60 µm Myc-I for the last 8 h before being harvested. Lysates were subjected to IP with Beclin 1 antibody (as described in the Methods section) and then assessed for Bcl2 levels by western blotting. Arrow indicates Bcl2 band.

To further investigate the molecular mechanism involved in Myc-modulated autophagy, we examined potential molecular players upstream of Bcl2. Activation of JNK1 has been reported to phosphorylate Bcl2, thereby inducing autophagy in cells under starvation conditions (6). Hence, we examined the role of JNK1 in Myc-dependent Bcl2 phosphorylation and autophagy. Specifically, we studied whether the changes in Bcl2 phosphorylation was a consequence of changes in JNK1 activity. We observed that Myc knockdown led to a reduction in JNK1 phosphorylation, and this occurred in parallel with decreased phosphorylation of Bcl2 (Fig. 5A). Correspondingly, Myc-I treatment decreased the amount of JNK1 being phosphorylated (Fig. 5B). Consistent with the literature (35), LC3-II levels were found to be lower in HeLa cells treated with a JNK inhibitor (JNK-Inh), and this was associated with decreased phospho-Bcl2 (Supplementary Material, Fig. S4A). JNK-Inh also decreased LC3-II levels in Myc WT rat fibroblasts (Fig. 5C). JNK-Inh further lowered LC3-II levels even in Myc −/− fibroblasts, suggesting that JNK1 acts downstream of Myc in modulating autophagy. To confirm the hypothesis that JNK1 is downstream of Myc in autophagy regulation, JNK1 knockout (−/−) and its control (JNK +/+) MEFs were used. Myc-I could decrease LC3-II levels in JNK +/+ MEFs, but this effect was abolished in JNK −/− MEFs (Fig. 5D). In addition, we showed that transient overexpression of constitutively active JNK1 in HeLa cells could rescue the levels of LC3-II that would otherwise be decreased in Myc knockdown cells, suggesting that constitutive JNK1 could abolish the effect of Myc depletion on autophagy, perhaps by overriding the decreased JNK phosphorylation resulting from Myc knockdown (Supplementary Material, Fig. S4B). These data supported the notion that Myc depletion led to autophagy impairment through decreased JNK activity and phosphorylation of its downstream target, Bcl2.

Phosphorylation of Bcl2 by JNK1 has previously been shown to result in dissociation of Bcl2 from Beclin 1, thereby relieving Beclin 1 from its inhibitory interaction with Bcl2 and allowing Beclin 1 to activate autophagy via Vps34 (6). We therefore tested whether the decrease in autophagy observed with Myc inhibition is associated with altered Bcl2-Beclin interactions. Consistent with decreased autophagy mediated through a reduction in phospho-Bcl2 and phospho-JNK1, Myc inhibition increased the interaction between Bcl2 and Beclin 1 (Fig. 5E).

In addition, as activation of JNK1 has been shown to induce autophagy under starvation conditions, we went on to investigate if Myc inhibition has the same effect on autophagy during starvation. While starvation clearly induced autophagy compared to nutrient-replete full medium, it did not increase the number of GFP-LC3 dots in cells treated with Myc-I (Supplementary Material, Fig. S4C). The extent of autophagy inhibition caused by Myc-I treatment in starvation conditions appears to be greater than the magnitude of autophagy inhibition in full medium condition because starvation is capable of increasing autophagy. Similar to Myc-I, treatment with JNK-Inh failed to trigger starvation-induced autophagy, consistent with the notion that the effects of Myc inhibition on autophagy are mediated through JNK activation.

JNK1 is a member of the MAPK family which is activated by various stress signals, including oxidative stress, UV irradiation and injury stress associated with pro-inflammatory cytokines (36). Thus, we also examined the effects of Myc suppression on other members of the MAPK family, including p38 MAPK and ERK. However, we did not observe any significant changes in the phosphorylation levels of p38 MAPK or ERK after Myc knockdown (Supplementary Material, Fig. S5A).

**Figure 6.**
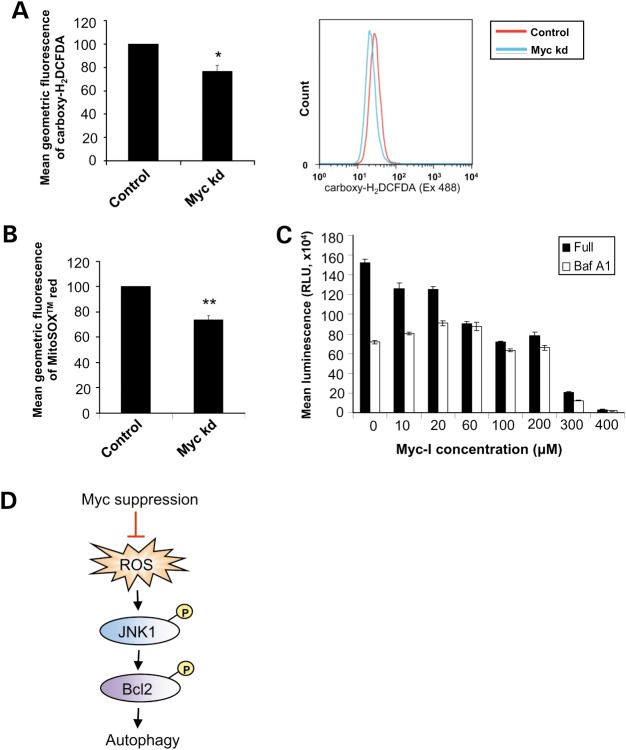
ROS levels were lower in MYC knockdown cells. HeLa cells subjected to control or Myc siRNA knockdown for 72 h were assessed for total ROS levels by FACS analysis of (**A**) carboxy-H_2_-DCFDA (a representative histogram of carboxy-H_2_-DCFDA fluorescence measurement by FACS analysis is shown); and (**B**) MitoSox™ red fluorescence. Graphs show mean ± SEM of mean geometric fluorescence of three independent studies, in which the control condition has been set to 100. *P < 0.05; **P < 0.005. (**C**) HeLa cells treated with 200 nm Baf A1 were co-treated with different concentrations of Myc-I as indicated for 16 h. Cells were assessed for viability with CellTiter-Glo luminescent assay (as described in the Methods section). Graph shows mean ± SEM of mean luminescence (in relative fluorescence unit, RLU) from a triplicate experiment representative of three independent studies. (**D**) Schematic illustrating the pathway involved in regulation of autophagy resulting from Myc depletion: Myc suppression is associated with decreased ROS production, which leads to reduced phosphorylation of JNK1 and its downstream target, Bcl2, thereby inhibiting autophagy.

In addition, recent work has demonstrated that the superoxide-inducing agent, menadione, strongly enhanced autophagy (37). As oxidative stress related to reactive oxygen species (ROS) signalling has been known to regulate autophagy (38,39), and Myc is known to induce ROS accumulation, we therefore examined the role of ROS in modulation of autophagy resulting from Myc suppression. We found that Myc knockdown cells contained lower levels of ROS in assays with 6-carboxy-2’,7’-dichlorodihydrofluorescein diacetate (carboxy-H_2_DCFDA) or MitoSOX™ red (Fig. 6A and B). Consistent with this, treatment with hydrogen peroxide nullified the effect of Myc knockdown on autophagy and restored LC3-II levels comparable with controls (Supplementary Material, Fig. S5B).

Inhibition of Myc has been shown to trigger cell growth arrest and apoptosis in transformed cells as well as tumour xenografts (18–20). Thus, we examined the consequences of autophagy-lysosome inhibition in the context of Myc suppression inhibition on cancer cell survival (Fig. 6C) by assessing the effects of increasing dosage of Myc-I on cell viability in the absence or presence of Baf A1. Myc-I clearly decreased cell viability compared with control. The extent of reduction in cell viability in response to Myc-I treatment was dose-dependent without Baf A1. The presence of Baf A1 consistently resulted in a lower cell viability compared with cells without Baf A1 at lower concentrations of Myc-I. Intriguingly, when autophagy was inhibited with Baf A1 treatment, the presence of Myc-I only minimally decreased cell viability from 0 to 60 µm. Our data indicate that autophagy-lysosome attenuation with Baf A1 decreased cell viability itself and delayed the onset of response towards Myc-I in terms of inhibition of cell viability. This also implies that the effects of Myc inhibition on cell viability were at least partially dependent on the autophagy-lysosome pathway.

Collectively, our data suggest that Myc knockdown, through decreased generation of ROS, resulted in less phosphorylation of JNK1 and Bcl2, which consequently led to a reduction in autophagy (Fig. 6D).

## DISCUSSION

Myc plays important roles in many fundamental aspects of cell biology, such as cell proliferation, differentiation and sensitivity to apoptosis (40). Our study identifies a novel function of Myc in autophagy, a process important for the maintenance of cellular homeostasis. Myc knockdown impaired autophagy through decreased phosphorylation of JNK1 and its downstream target Bcl2. The functional relevance of this observation reinforces the attractiveness of targeting Myc as a therapeutic strategy for cancers, since autophagy promotes cell survival under stress conditions, such as nutrient depletion and hypoxia, often encountered by established tumour cells.

Previously, the anti-neoplastic effect of Myc inactivation has been associated with mechanisms such as proliferative arrest, cellular senescence, terminal differentiation and apoptosis induction (18,19). None has directly linked the effects of Myc depletion with decreased autophagy. In fact, earlier studies have shown that cancer cells have decreased autophagic activity and increased accumulation of the autophagic substrates p62 (41). In addition, tumour suppressors such as Beclin 1, PTEN and DAP kinase are known to positively regulate autophagy (42). Therefore, one would expect an oncogene to have inhibitory effects on autophagy. Following this, Myc depletion would be expected to induce autophagy. On the contrary, we showed that Myc knockdown decreased autophagy. This is further confirmed by inhibition of Myc with small molecule inhibitors, 10058-F4 and 10074-G8, which target different regions of Myc, and both resulted in impaired autophagy as well as decreased Bcl2 activity.

Previously, autophagy inhibition with chloroquine has been demonstrated to promote apoptosis and tumour regression in a Myc-induced lymphoma model (43). The study suggested that autophagy is an adaptive response to enhance the survival of tumour cells in the face of apoptotic insults. It was therefore proposed that apoptosis activators can potentially be used in combination with autophagy inhibitors to enhance its therapeutic effects in human cancers. Consistent with their data, we found that inhibition of autophagy with Baf A1 decreased cell viability compared with cells without treatment. In addition, we showed that treatment with Myc-I, which inhibits autophagy, reduced cell viability in a dose-dependent manner. Myc presents an attractive target for cancer therapy as Myc suppression by different strategies has been shown to induce cell growth arrest and apoptosis in transformed cells, as well as tumour regression in Myc-induced cancers (18,19). In light of our data presented here, work by Amaravadi and colleagues lends further support to targeting Myc as an anti-neoplatic strategy, especially in Myc-induced tumours, as this might confer the additional benefit of inhibiting autophagy in the case of therapy resistance associated with chemotherapy-induced autophagy. Furthermore, our data suggest that one of the added benefits of Myc inhibition in the tumour context is autophagy compromise.

Elucidation of the molecular mechanisms involved in Myc regulation of autophagy is important for understanding of the feasibility and potential side effects that could arise from inactivation of Myc and its affected downstream targets. The closest observation was made by Tsuneoka and colleagues using a stable inducible expression of the MycER system in rat 3Y1 fibroblasts (44), whereby they reported that the overexpression of Myc was correlated with increased autophagic vacuoles. However, increase in autophagic vacuoles (or LC3-II) does not necessarily reflect an increase in autophagic flux–it may be a result of a block in the autophagosome degradation step, thus leading to an accumulation of autophagosomes. In addition, overexpression of Myc to non-physiological levels may lead to artefacts and confounding results. Different from their approach and in line with the relevance of Myc inactivation as an anti-neoplastic strategy, we showed that Myc knockdown decreases autophagosome formation. We also elucidated JNK1 and Bcl2 phosphorylation as plausible effectors involved in the regulation of autophagy by Myc suppression, and that this is likely mediated through changes in downstream Bcl2–Beclin1 interactions.

In addition to the plausible pathway that we have characterized in this study, we cannot exclude the possibility that there are other indirect pathways involved as well. Although decrease in ROS is expected to affect other members of the MAPK family, p38 MAPK and ERK were not significantly affected by Myc suppression, perhaps due to other compensatory mechanisms caused by changes in Myc activity (45). Nonetheless, our data indicate that regulations of JNK1 and Bcl2 are key consequences impacting on autophagy that result from Myc inhibition. In conclusion, our data provide mechanistic insights not only into the association between Myc and autophagy, but also into the functional relevance that Myc suppression has in the development of potential cancer therapy.

## MATERIALS AND METHODS

### Cell culture

HeLa cells, HEK293 cells, WT TGR-1 (Myc WT) and c-Myc null HO15.19 (Myc −/−) rat fibroblast cell lines (generous gift of J.M. Sedivy) (46), Atg5 −/− MEFs (kindly provided by N. Mizushima) (47) , and JNK1 −/− MEFs (kind gift of R.J. Davies) (48) were grown in Dulbecco's Modified Eagle's Medium (DMEM, Sigma) supplemented with 10% Fetal Bovine Serum (FBS, Sigma), 2 mm l-glutamine and 100 U/ml penicillin/streptomycin at 37°C in 5% CO_2_. HeLa cells stably expressing the GFP-LC3 (kind gift of A.M. Tolkovsky) (49) and HEK293 stably expressing GFP-DFCP1 (kindly provided by N.T. Ktistakis) (28) were cultured in a similar medium containing 500 µg/ml G418. Stable inducible GFP-p62 HEK293 cells (from T. Johansen) (50) were cultured as above, with the addition of 100 µg/ml hygromycin B (Calbiochem) and 7.5 µg/ml blasticidin (Invitrogen) to the culture medium.

Cells were seeded 24 h before transfection such that they reached 70–80% confluency the next day. cDNA constructs or siRNA were transfected using Lipofectamine 2000 reagent (Invitrogen) in OptiMEM (Invitrogen) by following the manufacturer's protocols. pcDNA3.1 empty vector was used as a control in overexpression studies, whereas nontargeting siRNA was used in siRNA experiments (SMARTpool; Thermo Fisher Scientific).

### DNA constructs

The cDNA constructs used in transfection were obtained from the following sources: pcDNA3-c-Myc from W. El-Deiry (Addgene plasmid 16011) (51), and Flag-CA JNK1 (Flag-MKK7B2Jnk1a1) from R.J. Davis (Addgene plasmid 19726) (51). Dendra-tau was obtained by subcloning WT human MAPT (2N4R) in pcDNA3.1 (a gift from L. Gan) (52) into pDendra2 vector (Evrogen) at KpnI and ApaI sites. Beclin 1 and Bcl2 plasmids were described earlier (53).

### Reagents

All chemicals used in this study were dissolved in dimethyl sulfoxide (DMSO) unless otherwise stated. The following chemical compounds were used for treatment at the following concentrations: bafilomycin A1 (400 nm, Millipore), 10058-F4 (60 µm, Calbiochem), 10074-G5 (30 µm, Sigma), HA14-1 (20 µm, Sigma), JNK inhibitor VIII (20 µm, Calbiochem), Carboxy-H_2_DCFDA (20 µm, Invitrogen) and MitoSOX™ red (5 µm, Invitrogen).

Primary antibodies used were: rabbit anti-c-Myc (diluted at 1:1000, Santa Cruz), rabbit anti-LC3 (diluted at 1:2000, Novus Biologicals), rabbit anti-actin (diluted at 1:2000, Sigma), mouse anti-α-tubulin (diluted at 1:5000, Sigma), mouse anti-GFP (diluted at 1:5000, Clontech), rabbit anti-phospho-Bcl2 (diluted at 1:1000, Cell Signalling), rabbit anti-Bcl2 (diluted at 1:1000, Santa Cruz), rabbit anti-phospho-JNK (diluted at 1:1000, Cell Signalling), mouse anti-JNK (diluted at 1:1000, Santa Cruz), rabbit anti-Beclin1 (diluted at 1:1000, Cell Signalling), mouse anti-p62 (diluted at 1:1000, BD transduction Laboratories), rabbit anti-phospho-S6K (diluted at 1:1000, Cell Signalling), rabbit anti-S6K (diluted at 1:1000, Cell Signalling), rabbit anti-phospho-S6 (diluted at 1:1000, Cell Signalling) and rabbit anti-S6 (diluted at 1:1000, Cell Signalling).

### Western blot analysis

Assessment of protein levels was carried out by standard sodium dodecyl sulphate–polyacrylamide gel electrophoresis (SDS–PAGE) and western blotting techniques. In brief, cells were lysed in radioimmunoprecipitation assay (RIPA) buffer [150 mm sodium chloride, 1% (w/v) sodium deoxycholate, 50 mm Tris (pH 7.4), 1% (v/v) Triton X-100, 0.25 mm EDTA, 5 mm sodium orthovanadate, 50 mm sodium fluoride, phosphatase inhibitor and protease inhibitor (Roche Molecular Biochemicals)] and the supernatant was collected for analysis by immunoblotting. To ensure equal loading of samples, protein concentration was determined by using a DC protein assay (Biorad) before being subjected to a SDS–PAGE.

Blots were probed with primary antibodies overnight followed by horseradish peroxidase-conjugated anti-mouse or anti-rabbit IgG (Roche) at 1:5000. Protein bands were detected using an enhanced chemiluminescence (ECL) western blotting kit (GE Healthcare) and densitometry quantified using ImageJ software (National Institutes of Health).

### Autophagy assesment by automated cellomics microscope

HeLa cells stably expressing GFP-LC3 were cultured on coverslips and subjected to siRNA knockdown or drug treatment. They were then rinsed once with phosphate-buffered saline (PBS) and fixed with 4% paraformaldehyde, followed by mounting of coverslips on slides using Prolong Gold antifade reagent with 4’, 6-diamidino-2-phenylindole (Invitrogen). The number of GFP puncta per cell was quantified with the Spot detector V3 Cellomics Bioapplication in a Thermo Scientific Cellomics ArrayScan High Content Screening Reader. At least 300 cells were scored for each sample and the average number of dots per cell was computed by the ArrayScan software.

### HaloTag-p62 clearance assay for autophagy activity

U2OS cells stably expressing HaloTag-p62 (construct was obtained from Kazusa DNA Research Institute collection) (previously described in (54)) were cultured in DMEM supplemented with 10% FBS (Sigma), 2 mm l-glutamine, 100 U/ml streptomycin/penicillin and 100 µg/ml hygromycin B at 37 °C, 5% CO_2_. To assay for p62 clearance, cells were seeded in 12-well plates and the next day, they were labelled with a diAcFam-conjugated fluorescent HaloTag ligand (Promega) according to manufacturer's instructions. Labelled cells were subsequently treated with Myc-I (60 µm) or DMSO as a control for 16 h before being harvested as described above and subjected to SDS–PAGE. Gels were scanned and fluorescence was measured with a Typhoon 8600 imager. For quantification of total protein loading, Krypton Flourescent Protein Stain (Pierce) was used following the manufacturer's instructions. Fluorescence levels were quantified by densitometric analysis using Image J software.

### Dendra-tau clearance assay

Dendra-tau was transfected into HeLa cells with Lipofectamine 2000. After 24 h, Dendra-tau was either photoconverted from green to red by exposure of cells to a 405 nm LED light source (constructed in-house) for 2 min or left unconverted (as controls). Both converted and unconverted cells were subjected to control or Myc siRNA knockdown for 48 h before being fixed and analysed by fluorescence-activated cell sorting (FACs) for Dendra-tau levels. The red fluorescent signal was assessed at excitation and emission wavelengths of 561/586 using a FACS analysis BD LSR Fortessa cell analyser (BD Biosciences). For compound treatment, cells were similarly photoconverted prior to treatment with the various drugs for 16 h before being analysed by FACS.

### Immunoprecipitation

Cells collected for immunoprecipitation (IP) study were lysed in Buffer A (20 mm Tris–HCl, pH 7.4, 2 mm MgCl_2_, 150 mm NaCl, 5 mm NaF, 1 mm Na_3_VO_4_, 0.5% NP-40, protease inhibitor cocktail (Roche)) for 20 min on ice, followed by centrifugation at 13 000*g* for 15 min. Cell lysates were incubated with an anti-Beclin 1 antibody (Cell Signalling) overnight at 4°C, and subsequently with Dynabeads Protein G (Invitrogen) for 3 h at 4°C. Products of IP were boiled in Laemmli buffer and analysed by western blotting.

### ROS assay

HeLa cells were monitored for intracellular ROS generation using a peroxide-sensitive fluorescent probe, 2′, 7′-dichlorofluorescin diacetate (Carboxy-H_2_DCFDA); or mitochondrial superoxide sensitive dye, MitoSOX™. After 72 h of siRNA knockdown, cells were washed once with PBS and incubated with phenol red-free complete medium (Gibco) containing 20 µm carboxy-H_2_DCFDA for 20 min or 5 µm MitoSOX™ red (Invitrogen) for 10 min at 37°C. Subsequently, cells were harvested with trypsin and washed twice with phenol red-free medium. Following this, they were resuspended in phenol red-free medium. The fluorescent signal was assessed at excitation and emission wavelengths of 488/530 using a FACS Calibur flow cytometer (Beckman Coulter, California, USA) for carboxy-H_2_DCFDA and using a BD LSR Fortessa cell analyser (BD Biosciences) for MitoSOX™ red. At least 10 000 cells were measured for each sample. Data were analysed using the FlowJo software (Tree Star, Inc.) and presented as the geometric mean of green fluorescence for carboxy-H_2_DCFDA or red signal for MitoSOX™ red.

### Cell viability assay

Cell viability was determined by using a CellTiter-Glo Luminescent Cell Viability Assay kit (Promega) following the manufacturer's instructions. Briefly, 100 µl of Cell Titer-Glo reagent was added to cells grown in 100 µl of culture medium in a 96-well plate. The contents were mixed on a shaker for 5 min and incubated at room temperature for 10 min. Luminescence was then measured with a GloMax luminometer (Promega).

### Statistical analyses

Immunoblots were quantified by densitometric analysis with the Image J program (Rasband, W.S., ImageJ, US National Institutes of Health, Bethesda, MD, USA, http://rsb.info.nih.gov/ij/, 1997–2005). Protein signals were normalized to actin as loading control. The *P*-values were determined by two-tailed Student's *t*-test in at least three independent experiments. Statistical significance for comparisons between more than two groups was analyzed by one-way ANOVA with a Dunnett or Bonferroni multiple comparisons *post hoc* test in PRISM software.

## SUPPLEMENTARY MATERIAL

Supplementary Material is available at *HMG* online.

*Conflict of Interest statement*. None declared.

Supplementary Data
