# Supplementary material for: Myc inhibition impairs autophagosome formation
Source: Hum Mol Genet. 2013 Aug 9;22(25):5237–48. doi: 10.1093/hmg/ddt381 (PMC3842180; doi:10.1093/hmg/ddt381)
Supplement: Supplementary Data [file supp_22_25_5237__index.html]

Myc inhibition impairs autophagosome formation — Myc inhibition impairs autophagosome formation — Supplementary Data 

# Myc inhibition impairs autophagosome formation

## Supplementary Data

Supplementary Data

**Files in this Data Supplement:**

- Supplementary Data - Pdf file
