# Supplementary material for: Myc inhibition impairs autophagosome formation
Source: Hum Mol Genet. 2013 Aug 9;22(25):5237–48. doi: 10.1093/hmg/ddt381 (PMC3842180; doi:10.1093/hmg/ddt381)
Supplement: Supplementary Data [file supp_ddt381_ddt381supp.pdf]

Supplementary Material for

Myc inhibition impairs autophagosome formation

Pearl P. C. Toh, Shouqing Luo, Fiona M. Menzies, Tamás Raskó, Erich E. Wanker and David C. Rubinsztein*

*To whom correspondence should be addressed. Email: dcr1000@cam.ac.uk

This file includes:

1. Supplementary Figures (Fig. S1-S6)
2. Supplementary Figure Legends

1. SUPPLEMENTARY FIGURES

FIGURE S1

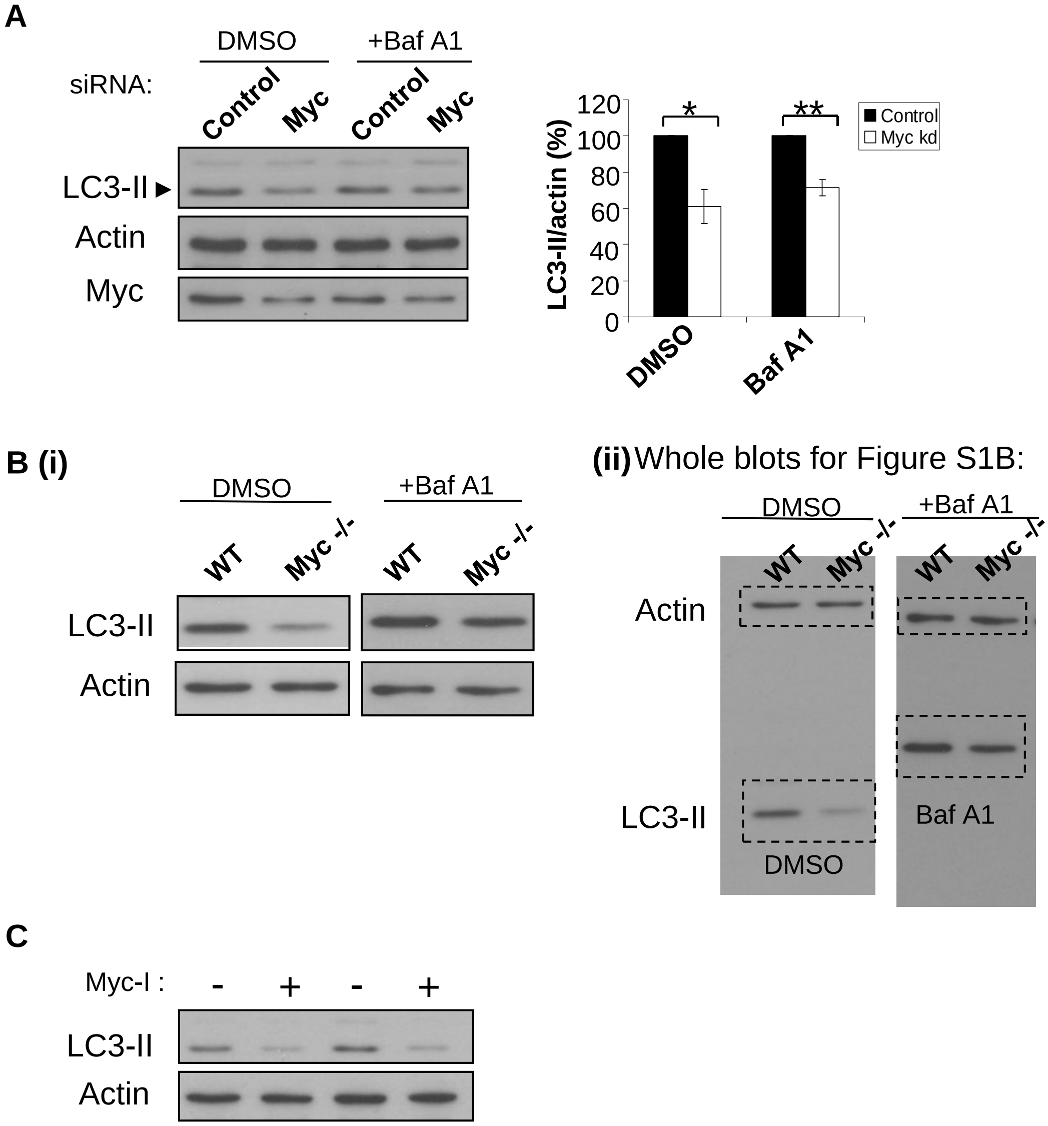

FIGURE S2

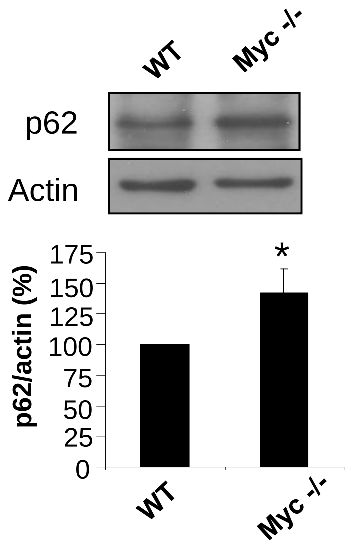

FIGURE S3

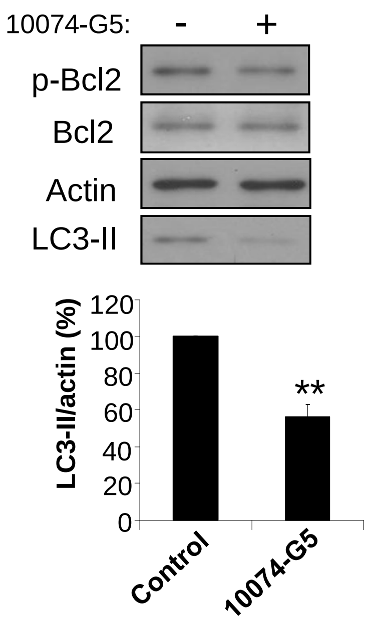
10074-G5:
-
+
p-Bcl2
Bcl2
Actin
LC3-II
LC3-II/actin (%)
120
100
80
60
40
20
0
**
Control
10074-G5

FIGURE S4

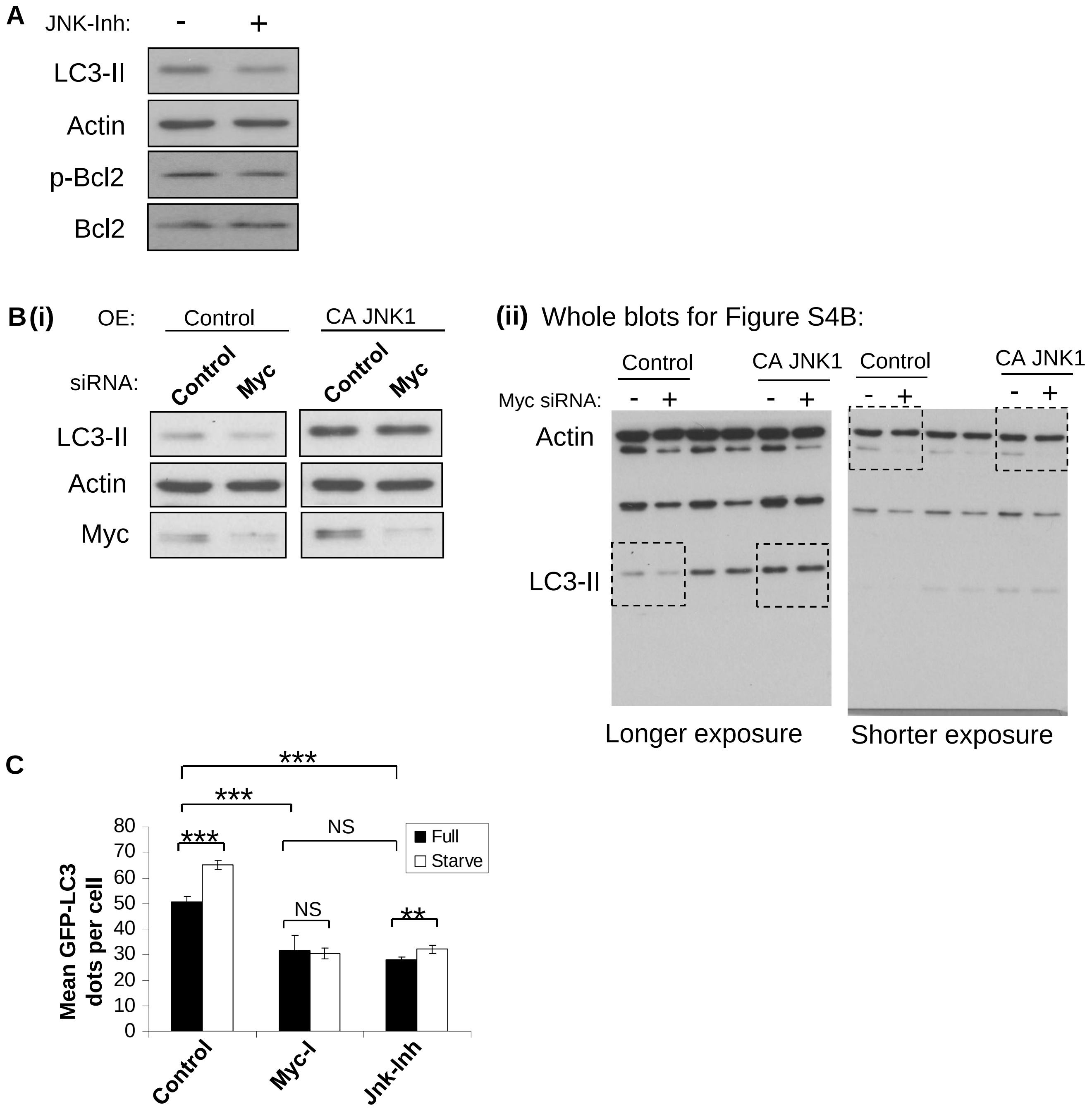
A
JNK-Inh:
-
+
LC3-II
Actin
p-Bcl2
Bcl2
B (i)
OE:
Control
CA JNK1
siRNA:
Control
Myc
Control
Myc
LC3-II
Actin
Myc
(ii) Whole blots for Figure S4B:
Control
CA JNK1
Control
CA JNK1
Myc siRNA:
-
+
-
+
-
+
-
+
Actin
LC3-II
Longer exposure
Shorter exposure
C
***
***
***
NS
NS
**
Full
Starve
Mean GFP-LC3 dots per cell
0
10
20
30
40
50
60
70
80
Control
Myc-I
Jnk-Inh

FIGURE S5

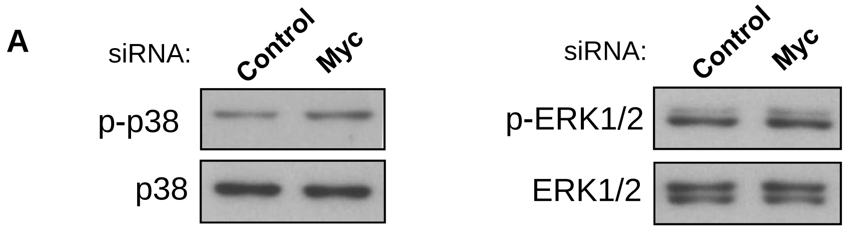
A
siRNA:
Control
Myc
p-p38
p38
siRNA:
Control
Myc
p-ERK1/2
ERK1/2

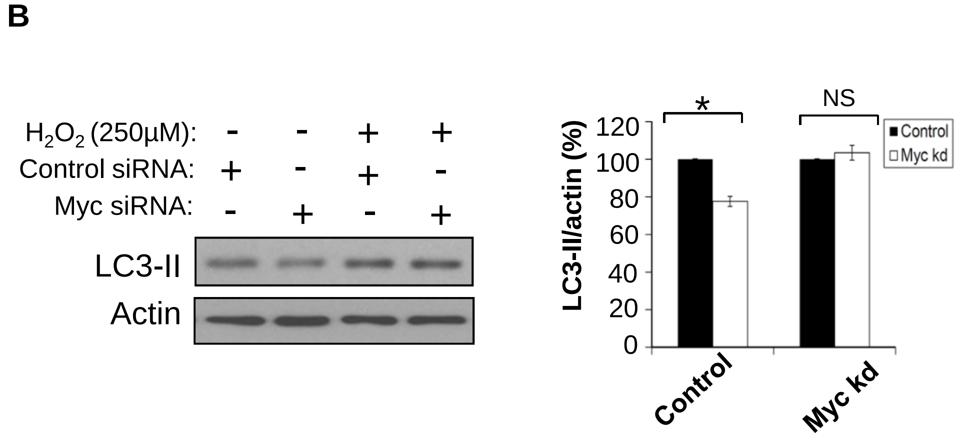
B
H2O2 (250µM): - - + +
Control siRNA: + - + -
Myc siRNA: - + - +
LC3-II
Actin
*
NS
Control
Myc kd
LC3-II/actin (%)
120
100
80
60
40
20
0
Control
Myc kd

FIGURE S6

Whole blots for Figure 5D:

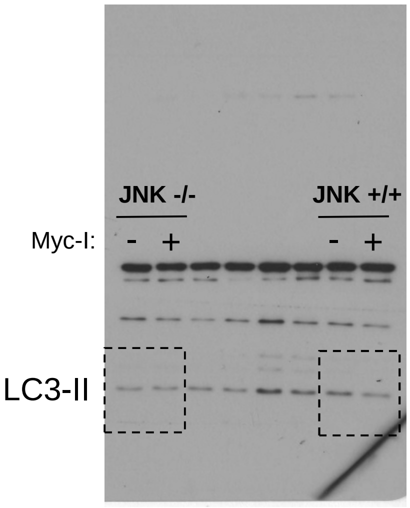

Longer exposure

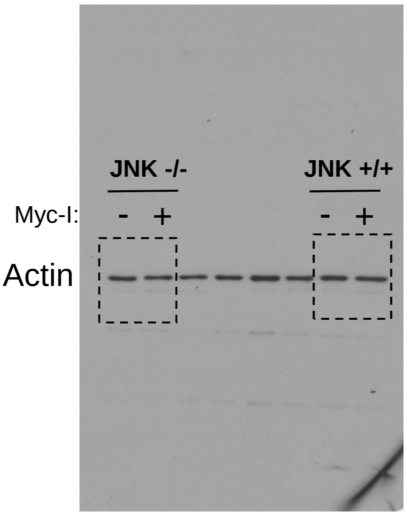

Shorter exposure

2. SUPPLEMENTARY FIGURE LEGENDS

Figure S1

(A) HEK293 cells subjected to control or Myc siRNA knockdown for 72 h were treated with 400 nM bafilomycin A1 (Baf A1) or DMSO during the last 4 h. Cells were lysed and assessed for LC3-II levels by western blotting. Graph shows quantification of LC3-II/actin ratio. The values represent the mean ± SEM of the percentage of LC3-II/actin from three independent experiments. $^{*}P<0.05$; $^{**}P<0.005$ by two-tailed Student's t-test as described in methods.

(B) (i) Myc+/+ (WT) or Myc-/- rat fibroblasts treated with 400 nM Baf A1 or DMSO for 4 h were assessed for LC3-II levels by western blotting. **(ii)** Whole blots for (i).

(C) HEK293 cells treated with 60 µM Myc inhibitor (Myc-I) for 8 h were assessed for LC3-II levels by western blotting.

Figure S2

Myc+/+ (WT) or Myc-/- rat fibroblasts were probed for p62. Graph shows quantification of p62/actin ratio. The values represent the mean ± SEM of the percentage of p62/actin from three independent experiments. $^{*}P<0.05$.

Figure S3

HeLa cells treated with 30 µM of another Myc inhibitor (10074-G5) for 16 h were assessed for phospho-Bcl2 and LC3-II levels by western blotting. The values represent the mean ± SEM of the percentage of LC3-II/actin from three independent experiments. $^{**}P<0.005$.

Figure S4

(A) HeLa cells treated with 20 µM JNK inhibitor VIII (JNK-Inh) for 3 h were assessed for phospho-Bcl2 and LC3-II levels by western blotting.

(B) (i) HeLa cells subjected to control or Myc siRNA knockdown for 72 h were transfected with pcDNA3.1 empty vector, or constitutive active (CA) JNK1 overexpression constructs for the last 24 h. Cell lysates were assessed for LC3-II levels by western blotting. **(ii)** Whole blots for (i).

(C) HeLa cells stably expressing GFP-LC3 treated with either 60 µM Myc-I for 16 h or 20 µM JNK-Inh for 3 h were cultured under full medium or 3 h HBSS starvation

condition before being fixed for quantification of LC3 dots. The number of LC3 dots was quantified by automated counting (see methods). At least 300 cells were counted per sample. Graph shows quantification of the mean of GFP-LC3 dots per cell. The values represent the mean ± SEM of the mean of GFP-LC3 dots per cell whereby ≥300 cells per sample were analysed in a study representative of three independent experiments. ***P<0.0005; **P<0.005, by one-way ANOVA, with Bonferrroni post hoc test. ***P<0.0005 for control vs. treatment pair (with either Myc-I or JNK-Inh) in each full medium condition and starvation condition.

Figure S5

(A) HeLa cells subjected to control or Myc siRNA knockdown for 72 h were assessed for phospho- or total p38 (left panel) or phospho- or total ERK1/2 (right panel) by western blotting.

(B) HeLa cells subjected to control or Myc siRNA knockdown for 72 h were treated with 250 µM H_2O_2 for the last 24 h. Cell lysates were assessed for LC3-II levels by western blotting. Graph shows quantification of LC3-II/actin ratio. The values represent the mean ± SEM of the percentage of LC3-II/actin from three independent experiments. *P<0.05; NS, not significant.

Figure S6

Whole blots for Figure 5D.
